# Subjective Socioeconomic Status, Cognitive Abilities, and Personal Control: Associations With Health Behaviours

**DOI:** 10.3389/fpsyg.2021.784758

**Published:** 2022-01-28

**Authors:** Pål Kraft, Brage Kraft, Thomas Hagen, Thomas Espeseth

**Affiliations:** ^1^Department of Psychology, University of Oslo, Oslo, Norway; ^2^Oslo New University College, Oslo, Norway; ^3^Division of Psychiatry, Diakonhjemmet Hospital, Oslo, Norway

**Keywords:** health behaviours, income, education, subjective socioeconomic status, intelligence, executive functioning, personal control, mastery

## Abstract

**Objective:**

To examine subjective and objective socioeconomic status (SSES and OSES, respectively) as predictors, cognitive abilities as confounders, and personal control perceptions as mediators of health behaviours.

**Design:**

A cross-sectional study including 197 participants aged 30–50 years, recruited from the crowd-working platform, Prolific.

**Main Outcome Measure:**

The Good Health Practices Scale, a 16-item inventory of health behaviours.

**Results:**

SSES was the most important predictor of health behaviours (beta = 0.19, *p* < 0.01). Among the OSES indicators, education (beta = 0.16, *p* < 0.05), but not income, predicted health behaviours. Intelligence (*r* = −0.16, *p* < 0.05) and memory (*r* = −0.22, *p* < 0.01) were negatively correlated with health-promoting behaviours, and the effect of memory was upheld in the multivariate model (beta = −0.17, *p* < 0.05). Personal control perceptions (mastery and constraints) did not act as mediators.

**Conclusion:**

SSES predicted health behaviours beyond OSES. The effect of socioeconomic indicators was not confounded by cognitive abilities. Surprisingly, cognitive abilities were negatively associated with health-promoting behaviours. Future research should emphasise SSES as a predictor of health behaviours. Delineating the psychological mechanisms linking SSES with health behaviours would be a valuable contribution toward improved understanding of socioeconomic disparities in health behaviours.

## Introduction

This study examined how individuals’ socioeconomic status (SES) is related to health behaviours, which are further related to socioeconomic disparities in health. Specifically, we addressed whether subjective socioeconomic status (SSES) predicted health behaviours beyond the indicators of objective socioeconomic status (OSES). Previous research has demonstrated that the association between socioeconomic indicators and health behaviours tends to be attenuated when controlling for cognitive abilities. Therefore, we included several measures of cognitive abilities (intelligence, attention, and memory) in the analyses. We also examined whether perceptions of personal control (mastery and constraints) mediated various measures of SES, cognitive abilities, and health behaviours.

Socioeconomic status reflects the position that a person holds in a social and economic hierarchy (Galobardes et al., 2006a, 2007). In general, individuals with different levels of SES experience different levels of access to resources and opportunities; are exposed to different levels of threats, scarcity, and adversities; and often follow different developmental trajectories (Adler et al., 2000; Kraus et al., 2012). However, SES can be conceptualised and measured in various ways. For example, it can be measured by structural or area-level measures (such as the index for multiple deprivation or postal code) or by individual measures, which were the focus of the present study. Individual level measures of SES include OSES, which represents an individual’s absolute levels of resources such as income or education (Galobardes et al., 2006b; Pampel et al., 2010), and SSES, which reflects a person’s perception of their position in a socioeconomic hierarchy relative to others (Singh-Manoux et al., 2003). Additionally, many studies on social disparities in health have included measures of childhood SES (e.g., parental education or income) (Galobardes et al., 2006a), most often collected from self-reports or registries. The cumulative research reporting OSES disparities in health and longevity is extensive (Stringhini et al., 2017; Kivimäki et al., 2020), although there are variations in how specific OSES indicators relate to specific health outcomes (Galobardes et al., 2007). SSES is also a robust predictor of health outcomes, most often measured as perceived health (Adler et al., 2000; Präg et al., 2016; Cundiff and Matthews, 2017; Zahodne et al., 2018; Tan et al., 2020). Moreover, childhood SES is an important predictor of health throughout the life course (Poulton et al., 2002; Danese et al., 2009; Cohen et al., 2010; Conroy et al., 2010). In sum, regardless of how SES is operationalised, the general finding is that SES matters for health and longevity. Of note is the fact that although SES disparities in health and longevity are a pressing public health concern in many countries, they have not been mitigated over the last few decades (Marmot et al., 2010; Eikemo et al., 2016). Importantly, low versus high SES is associated with an increased risk for many diseases, independent of health behaviours (Stringhini et al., 2017; Petrovic et al., 2018). Nevertheless, research has demonstrated that a substantial proportion of SES disparities in health can likely be attributed to SES differences in health behaviours (Stringhini et al., 2011; Petrovic et al., 2018; Mackenbach et al., 2019).

Socioeconomic status differences are seen in most health behaviours, such as cigarette smoking, drug abuse, heavy alcohol drinking, diet, weight management, physical activity, safety behaviours (e.g., seat belt and sunscreen use), adherence to medicinal procedures, self-examination, and participation in screening and vaccination schemes (Bickel et al., 2014; Pepper and Nettle, 2014b; Petrovic et al., 2018). The literature describing OSES disparities in health behaviours is vast. For example, a literature search in *PsycInfo* performed in April 2021 combining the following search string yielded more than 185,000 hits: smoking OR drug abuse OR heavy alcohol drinking OR diet OR weight OR physical activity AND education OR income OR occupation OR SES. To illustrate further, the combination of smoking and education yielded nearly 52,000 hits; that of smoking and income, more than 17,000 hits; smoking and occupation, 2,500 hits; smoking and SES, almost 12,000 hits; and smoking and poverty, 6,900 hits. Although we did not perform a systematic and comprehensive literature search, the simple search results illustrate that the literature is vast and that there is large heterogeneity in the use of OSES indicators. In comparison, the research literature linking SSES with health behaviours is limited. For example, the following search string yielded 318 hits: perceived socioeconomic status OR subjective socioeconomic status AND smoking OR drug abuse OR heavy alcohol drinking OR diet OR weight OR physical activity, while another search string—perceived socioeconomic status, subjective socioeconomic status, and cigarette smoking—yielded merely 157 hits.

Education, income, and occupational prestige are the most often applied indicators of OSES in health research (Galobardes et al., 2006a). These indicators are objective because they assign people to positions in a social hierarchy, independent of their subjective perceptions (Hoebel and Lampert, 2018). Hence, the assessment of OSES is assumed to involve factual reports of resources and life circumstances that can be reported with limited top-down psychological influences, such as personality and mood (Tan et al., 2020). However, it has been shown that various empirical indicators used to measure OSES are redundant and sometimes only moderately intercorrelated (Geyer et al., 2006). Hence, OSES is perhaps better conceived of as a summary measure than as a theoretical construct (Edwards and Bagozzi, 2000). More specifically, while OSES is consistently reported to predict health behaviours (Stringhini et al., 2011; Bickel et al., 2014), specific OSES indicators, such as education and income, may differently reflect various personal characteristics and psychological mechanisms (such as cognitive abilities) (Laaksonen et al., 2008; Pampel et al., 2010).

While OSES indicators are considered objective measures of socioeconomic position, SSES reflects a person’s perception of their relative socioeconomic position within the society (Adler et al., 2000). SSES is understood as a person’s perceptions of access to power and resources and exposure to burdens and threats, relative to others in a social hierarchy. However, research has typically reported moderate correlations between indicators of OSES and SSES (Kraus et al., 2012; Hoebel and Lampert, 2018; Tan et al., 2020). Various explanations have been suggested for these findings. First, although levels of income, educational attainment, and occupational prestige are referred to when people are asked to judge their SSES, individuals may differ in the specific OSES indicator(s) they consider to be most relevant and important for their SSES. Second, different individuals may ascribe different qualitative assessments to the same OSES indicators (Adler et al., 2000). Third, the averaging hypothesis suggests that SSES represents a more accurate and dynamic assessment because it may consider an individual’s past, current, and prospective position and resources in the sociocultural environment, whereas OSES represents more static and current resources (Singh-Manoux et al., 2005). Finally, OSES and SSES may correlate only moderately because they are differently influenced by the situational or social context (Destin et al., 2017). For example, in a rich society with substantial inequality, the feeling of being poor and placed at a low level in the socioeconomic hierarchy may be particularly salient for self-perceptions and personal identity (Wilkinson and Pickett, 2009; Cheung and Lucas, 2016). In sum, this suggests that SSES may tap the subjective meaning of being at a low level in a social system beyond what is measured by indicators of OSES. Indeed, research has demonstrated that SSES predicts health and longevity beyond the indicators of OSES (Cundiff and Matthews, 2017; Demakakos et al., 2018; Tan et al., 2020). Moreover, the perception of being at a low level in a socioeconomic hierarchy uniquely predicts various psychological and social outcomes (Cundiff and Matthews, 2017; Sheehy-Skeffington, 2018; Sherman and Mehta, 2019). Nevertheless, as we illustrated above, while research on the relationship between OSES and health behaviours has been abundant, comparatively less attention has been devoted to revealing the relationships between SSES and health behaviours. Despite such an imbalance in research focus, there have been reports of SSES predicting health compromising behaviours (Senn et al., 2014), smoking and quitting behaviour (Reitzel et al., 2014), physical activity (Frerichs et al., 2014), and diet (Wijayatunga et al., 2019). Quon and McGrath (2014) reported in a meta-analysis of 20 studies of adolescents that SSES correlated with some health behaviours (e.g., diet and physical activity) but not with others (e.g., substance-related health behaviours). While some of the studies included in the meta-analysis demonstrated that SSES predicted health behaviours beyond OSES, most did not report on the relative importance of OSES versus SSES in a multivariate model. Consequently, examining how SSES coordinates with various OSES indicators in predicting health behaviours would be a valuable contribution to improved understanding of socioeconomic disparities in health behaviours.

Research has demonstrated that the associations between SES and health behaviours tend to substantially weaken or become non-significant when controlling for cognitive abilities (Ciarrochi et al., 2012; Deary et al., 2019). Cognitive abilities are the skills involved in performing tasks associated with perception, learning, memory, understanding, awareness, reasoning, judgement, intuition, and language (APA, 2020). Cognitive abilities comprise two umbrella constructs with prominent similarities: executive functioning (EF) and intelligence (Duggan and Garcia- Barrera, 2015). EF is considered to bolster various cognitive processes, such as reasoning, problem-solving, and planning (Diamond, 2013). EF resources are also drawn on when we cannot behave automatically but must effortfully and deliberately hold information in mind to regulate goal-directed behaviour (Blair, 2016), that is, when we execute conscious self-control that helps us to override impulses and intentionally stop or activate ourselves in the service of higher order goals (Baumeister et al., 1994; Hofmann and Kotabe, 2012). A strong correlation and genetic overlap exist between EF and (fluid) intelligence (Engelhardt et al., 2015), which is the capacity to reason and solve novel problems independent of past knowledge, by identifying the patterns and relationships underpinning the given problems and solving them using logic (Cattell, 1963). Fluid intelligence is considered to underlie EF and all other types of cognitive abilities (Johnson et al., 2008).

A substantial amount of research has demonstrated socioeconomic disparities in cognitive abilities. On average, individuals with low-SES perform poorer on cognitive tests than those with high-SES (Noble et al., 2012; Farah, 2017). However, various studies have demonstrated that acutely induced resource scarcity causes functional changes in terms of poorer performance on EF tasks in all individuals, not only in those with low-SES (Liu et al., 2012; Mani et al., 2013). The suggested mechanism is that acute perceptions of scarcity and instability preoccupy cognitive resources and lead to worse performance on cognitive ability tests (Kurzban et al., 2013; Sheehy-Skeffington, 2019).

A recent systematic review provided an overview of 114 articles that examined the role of EF in predicting health behaviours (Reimann et al., 2020). The general finding was that lower EF was associated with more health-threatening behaviours and fewer health-promoting behaviours. However, a limitation of the review article was that it did not report effect sizes from the included studies. In contrast, such information was provided by Gray-Burrows et al. (2019) in the first comprehensive meta-analysis of studies on the relationship between EF and health behaviours in healthy populations. The results showed that EF was positively associated with health-promoting behaviours and negatively associated with health-threatening behaviours. However, the effect sizes were generally small (*r* = +0.090 and −0.044, respectively), and considerable heterogeneity existed among the studies. In summary, both the review and meta-analysis indicated that increased levels of EF were positively associated with healthy behaviours. However, it should be noted that a reciprocal relationship between cognitive abilities and health behaviours may exist. For example, physical activity seems to have a positive effect on EF (Allan et al., 2016), while drug abuse seems to have a negative effect (Brockett et al., 2018). Nevertheless, research on the effects of EF on health behaviours aligns with research on the relationship between intelligence and health behaviours. Hence, research in cognitive epidemiology demonstrates that intelligence is negatively related to smoking, binge drinking, poor diet, and physical inactivity (Ciarrochi et al., 2012; Wraw et al., 2018; Deary et al., 2019, 2021).

Thus, research has documented that cognitive abilities are associated with both SES and various health behaviours. However, to the best of our knowledge, the present study is the first to report how SSES coordinates with cognitive abilities in predicting health behaviours. We expand on previous research by measuring cognitive abilities through several tests, not limited to self-report measures, and by recruiting diverse participants through an online recruitment website, unlike many previous studies that restricted their sample to patients or students.

Furthermore, personal control was examined in the present study as a possible mediator of health behaviours. Personal control reflects beliefs about one’s ability to influence or control important life outcomes (Luszczynska and Schwarzer, 2005). When personal control is low, people feel less in control of being able to bring about positive events, avoid negative events, and achieve their goals (Luszczynska and Schwarzer, 2005). Most health behaviour theories include some aspects of personal control as determinants of health behaviours (Conner and Norman, 2015). Hence, people who believe that they have control over important elements of their lives are generally more likely to engage in health-promoting behaviours and less likely to engage in health-threatening behaviours (Norman et al., 1998; Luszczynska and Schwarzer, 2005; Pepper and Nettle, 2014a; Sheehy-Skeffington, 2018).

Research has shown that a person’s position in the social hierarchy is associated with perceptions of personal control (Ellis et al., 2009; Farah, 2017; Ishii et al., 2017; Sheehy-Skeffington and Rea, 2017). Regarding health behaviours, lower levels of personal control, in terms of both outcome and efficacy expectations, have been associated with performing less health-promoting behaviours and more health-threatening behaviours (Luszczynska and Schwarzer, 2005). There are indications that control beliefs (partially) mediate the association between OSES and health behaviours (Leganger and Kraft, 2003). However, no studies have examined whether SSES and cognitive abilities influence health behaviours via perceptions of personal control.

This study extends the previous research in three ways. First, we assessed the unique contribution of SSES and OSES indicators in predicting health behaviours. Second, we controlled for various types of cognitive abilities as potential confounders. Third, we examined whether personal control perceptions mediated between SSES, OSES, cognitive abilities, and health behaviours. Specifically, we hypothesised that (a) SSES would predict health behaviours beyond indicators of OSES; (b) the predictive effects of SSES and OSES on health behaviours would be attenuated when controlling for cognitive abilities (intelligence, attention, and memory); and (c) the predictive effects of SSES, OSES, and cognitive abilities, on health behaviours would be (partially) mediated via perceptions of personal control (mastery and constraints).

## Materials and Methods

### Participants

Participants were recruited via Prolific^1^, an online, crowd-working platform. Prolific participants meeting the following criteria were invited to participate in the study: age 30–50 years (as most people are likely to have finished their education by the age of 30 years), resident of the United Kingdom/British citizenship (to ensure homogenous reporting of education), and English as their first language (to ensure adequate comprehension of the questions). Due to financial constraints, we stopped recruitment when the sample size reached 100 men and 100 women. After a quality check of the responses, the final sample included 198 participants (99 women, 98 men, and one person who had a missing value for gender) with a mean age of 37.8 years (*SD* = 6.1 years).

Data on education, income, and SSES were obtained from the screening procedure that the participants underwent when they initially joined the Prolific community. Data on all other tests and variables were collected specifically for the present study. The average time for completing the tests and questions specifically set up for the present study was 57 min, and the compensation paid for participation was £8.32/60 min. We integrated several attention checks throughout data collection by including control items in several multi-item instruments. They were included to ensure that the participant had paid attention to the questions. For example, one control item was: “It’s important that you pay attention to this study.” Please tick “Strongly agree.” Individuals who failed one or more of the attention checks were excluded from further participation and replaced by new participants.

### Measures

*Health behaviours* were assessed using the *Good Health Practices Scale* (GHPS; Hampson et al., 2019). The GHPS is a 16-item inventory covering health behaviours, such as diet, exercise, smoking, weight control, self-examination, vaccination, medical check-ups, sleep, and dental behaviour. Each item is presented as a statement and responded to on a 5-point Likert scale, ranging from 1 (*not at all like me*) to 5 (*very much like me*). A compound score was calculated by summing and averaging the responses, with a higher score representing more health-promoting behaviours and fewer health-threatening behaviours (Cronbach’s α = 0.80).

In this study, we focused on individual measures of SES, rather than area-level measures (e.g., the index for multiple deprivation or postal code). *OSES* was measured by two indicators: personal income and highest education level completed. Personal income was measured by the question, ‘What is your personal income per year (after tax) in GBP?’ There were 11 response categories ranging from 1 (<£10,000) to 11 (>£150,000), which were recoded into five categories by merging some of the highest income categories that described only a few participants. Educational attainment was measured by the question, ‘Which of these is the highest level of education you have completed?’ There were seven response categories: no formal qualification; secondary education, e.g., GED/GCSE; A-levels/high school diploma; technical/community college; undergraduate degree, e.g., BA/BSc/other; graduate degree, e.g., MA/MSc/Mphil/other; and doctorate degree. The analysis showed that education and income were not significantly correlated (*r* = 0.14). Therefore, all the following analyses were conducted keeping income and education as separate variables.

*SSES* was assessed using the *MacArthur Scale of Subjective Social Status* – *Adult Version* (Adler et al., 1994), which measures how a person perceives their social standing. Respondents viewed a drawing of a ladder with 10 rungs and corresponding text explaining that the ladder represents where people stand in society. The text further read, ‘At the top of the ladder, are people who are the best-off, those who have the most money, most education, and best jobs. At the bottom, are people who are the worst-off, those who have the least money, least education, worst jobs, or no job. Please place an ‘X’ on the rung that best represents where you think you stand on the ladder.’ The MacArthur Scale was developed in order to capture individuals’ sense of their place in the socioeconomic hierarchy which takes into account their standing on multiple dimensions of socioeconomic status and social position. However, the scale does not directly address the subjective experience of for example poverty, exclusion, stress, unpredictability and lack of trust and fairness that is often related to living way down in the socioeconomic hierarchy (Haushofer and Fehr, 2014; Sheehy-Skeffington and Rea, 2017).

*Intelligence* was assessed using the *Hagen Matrices Test* (HMT), a free, web-based figural test focused on reasoning, to measure general intelligence (Amthauer et al., 2001; Heydasch, 2014; Heydasch et al., 2020). The original full test consists of three parts: instructions, 20 matrices, and presentations of the individual scores. Participants in this study completed an extended version of the short Hagen matrix test (HMT-S; Heydasch et al., 2017). In addition to the six standard items for the HMT-S, we added two items (items 10 and 14) from the full version of HMT (Heydasch, 2014), based on previous findings on the magnitude of correlations with the Intelligence Structure Test (I-S-T, 2000 R; Amthauer et al., 2001). Participants were instructed to complete 3 × 3 matrices with one missing field. Test takers had 2 min to choose one of eight presented alternatives, of which only one completes the matrix correctly. Eight items were presented following the instructions. A time counter informed participants about the amount of time that had passed for each item. If participants did not mark an answer within 2 min, the next item was presented. Correct answers were coded as 1, false or missing values were coded as 0, and a sum score was calculated.

The *EF* was measured using two tasks. First, we used the *multiple object tracking task* (MOT; Pylyshyn, 1989), measuring spatiotemporal attention capacity. In this task, 10 visually indistinguishable objects (balls) appeared on the screen. Thereafter, a subset of these objects (one to five targets) blinked briefly to designate them as target objects. Then, all objects started to move independently and unpredictably on the screen. The participants were instructed to track this subset of objects across a 10 s interval of object motion. The task started with a training round of six practice trials where participants were required to have at least three trials with 75% correctly tracked targets before continuing to the main experiment. After the training, the participants were presented with five blocks containing six trials each. Participants indicated the targets by clicking on them with the mouse pointer when the objects stopped moving. A compound score was calculated by summing and averaging the correct responses and was reported as the proportion of correct responses.

Additionally, EF was measured using a *spatiotemporal memory task* consisting of interleaving blocks of the learning and recognition phases. Similar to the MOT task, the stimuli consisted of balls moving on the screen. There were always three balls on the screen, and participants were instructed to remember the movement patterns. The learning phase consisted of four unique dynamic displays that were shown twice. In the subsequent recognition phase, all displays that were shown during learning were shown again (old), intermixed with an equal number of novel displays (new). The participants’ task was to make an old/new decision. The display consisted of three red balls on a grey background. The disks moved randomly on the square background for 5 s. The trajectories of the balls were randomised in the first and last second to remove the possibility of using the start and stop positions as memory cues. The experiment included 40 unique displays, each of which had an equal chance of being presented in the learning phase. These 40 displays were drawn from an initial sample of 500 displays based on a metric to be maximally different from each other in terms of the spatial locations of the stimuli (similar to the maximal Euclidian distance). We used the total accuracy (i.e., the ratio of correct to incorrect responses) to indicate the performance level.

*Perceived control* was assessed using the *Sense of Control Scale* from the *Midlife Development Inventory* (Lachman and Weaver, 1998)—a self-report measure of sense of control across two dimensions: perceived constraints (PC; eight statements) and personal mastery (PM; four statements). Responses were recorded on a 7-point Likert scale, ranging from 1 (*strongly disagree*) to 7 (*strongly agree*). PC measures locus of control and was measured by items such as, ‘Other people determine most of what I can and cannot do.’ PM measures general self-efficacy and was measured by items such as, ‘I can do just about anything I really set my mind to.’ Two compound scores were calculated by summation and averaging (PC: Cronbach’s α = 0.88; PM: Cronbach’s α = 0.80). Higher scores represent higher levels of PM and PC.

### Analysis

Descriptive statistics, correlation, and regression analyses were conducted using IBM SPSS Statistics version 26 (IBM SPSS Statistics for Windows, Version 26.0. Armonk, NY, United States: IBM Corp.). To test the mediation hypotheses, we used PROCESS for SPSS, a computational tool that can be used to analyse mediation with observed (manifest) variables and uses the ordinary least squares method (Hayes, 2013). Parameter estimates and 95% bias-corrected confidence intervals for direct and indirect effects were generated based on 10,000 bootstrapped samples.

## Results

Descriptive statistics are presented in Table 1, and bivariate correlations (Pearson’s *r*) between the measures are presented in Table 2. The correlation between the two OSES indicators of education and income (*r* = 0.14) was not statistically significant. However, both education (*r* = 0.26, *p* < 0.01) and income (*r* = 0.46, *p* < 0.01) positively correlated with SSES. Either education, nor income or SSES did correlate with any of the cognitive variables (intelligence, memory, and attention). Education (*r* = 0.21, *p* < 0.01) and SSES (*r* = 0.24, *p* < 0.01) were positively correlated with GHPS, meaning that those with higher education and SSES practiced more health-promoting behaviours and/or fewer health-threatening behaviours. In contrast, income did not correlate with GHPS. However, some of the items in the GHPS are potentially related to economic costs, while other items are not. Therefore, we constructed two sum-scores, one including health behaviours related to economic cost and the other including health behaviours that are not or are only minimally related to direct costs. However, none of the summed scores correlated with income.

**TABLE 1:** Descriptive statistics (*N* = 198).

Variables	Descriptive statistics					
	Possible range	Min	Max	*M*	*SD*	α
Education	1–7	1.00	7.00	4.65	1.36	
Income	1–5	1.00	5.00	3.10	1.61	
SSES	1–10	1.00	9.00	5.42	1.59	
Intelligence	0–8	0	7	3.95	1.71	
Memory	0–1	0.43	0.95	0.70	0.12	
Attention	0–1	0.59	0.95	0.80	0.08	
PC	1–7	1.00	7.00	3.33	1.18	0.88
PM	1–7	1.75	7.00	5.27	0.96	0.80
GHPS	1–5	1.50	4.56	3.00	0.57	0.80

*SD, standard deviation; α, Cronbach’s alpha.*

*SSES, subjective socioeconomic status; PC, perceived constraints; PM, perceived mastery; GHPS, good health practice scale.*

**TABLE 2:** Pearson’s correlation (*r*) between variables (*N* = 198).

	Correlation coefficients								
Variables	1	2	3	4	5	6	7	8	9
(1) Education		0.14	26**	0.09	−0.05	0.00	0.02	−0.05	0.21**
(2) Income			0.46**	−0.05	−0.10	−0.06	−0.22**	0.30**	−0.02
(3) SSES				−0.08	−0.06	−0.06	−0.22**	0.30**	0.24**
(4) Intelligence					0.41**	0.28**	0.15*	−0.10	−0.16*
(5) Memory						0.29**	0.07	−0.08	−0.22**
(6) Attention							0.08	−0.04	−0.04
(7) PC								−0.55**	−0.05
(8) PM									0.11
(9) GHPS									

*p < 0.05, **p < 0.01 (two-tailed tests).

*SSES, subjective socioeconomic status; PC, perceived constraints; PM, perceived mastery; GHPS, good health practice scale.*

The various measures of cognitive abilities were correlated, as expected. Hence, intelligence was positively correlated with memory (*r* = 0.41, *p* < 0.01) and attention (*r* = 0.28, *p* < 0.01). Attention and memory were also positively correlated (*r* = 0.29, *p* < 0.01). We hypothesised that we would observe positive correlations between intelligence, attention, and memory, and GHPS. However, while attention and GHPS did not correlate, negative correlations were observed between intelligence and GHPS (*r* = −0.16, *p* < 0.05), and between memory and GHPS (*r* = −0.22, *p* < 0.05). Hence, contrary to expectations, higher levels of intelligence and memory were related to performing less health-promoting and more health-threatening behaviours. Concerning perceptions of personal control, PC and PM were negatively correlated (*r* = −0.55, *p* < 0.01). PC was negatively correlated with income (*r* = −0.22, *p* < 0.01) and SSES (*r* = −0.22, *p* < 0.01), while PM was positively correlated with income (*r* = 0.30, *p* < 0.01) and SSES (*r* = 30, *p* < 0.01). Neither PM nor PC correlated with education or GHPS.

Variables that were significantly correlated with GHPS were included in the multivariate regression model. In this model, GHPS was regressed upon education, SSES, intelligence, and memory using the ordinary least-squares method. The multivariate model (Table 3) explained 13% of the total variance in GHPS. SSES (β = *0.19*, *p* < 0.01), education (β = *0.16*, *p* < 0.05), and memory (β = −0.17, *p* < 0.05) predicted GHPS, while the direct effect of intelligence did not reach statistical significance. Comparing the bivariate correlations and the predictive effects in the multivariate model, controlling for intelligence and memory attenuated, only slightly, the bivariate associations between education and SSES and GHPS.

**TABLE 3:** Multiple regression predicting GHPS (health behaviours) (*N* = 198).

Predictor	*B*	*SE* β	β
Education	0.07	0.03	0.16*
SSES	0.07	0.03	0.19**
Intelligence	−0.03	0.03	−0.09
Memory	−0.83	0.36	−0.17*
*R*^2^ = 0.13			

*p < 0.05, **p < 0.01.

*SSES, subjective socioeconomic status.*

Since PC and PM did not correlate with GHPS, we did not expect them to mediate any of the effects of the predictors of GHPS. It was previously considered that performing a mediation analysis was only sensible after one had established evidence of an association between mediator, predictor, and dependent variables. However, this no longer seems to be a precondition since indirect effects can occur in the absence of total effects (Zhao et al., 2010; Hayes, 2013). Therefore, separate mediation analyses were performed to examine the effects of education, SSES, and memory on GHPS. In each model, the relevant predictor was included along with GHPS as a dependent variable, PC and PM as potential mediators, and the other significant predictors as covariates (control variables). Parameter estimates and 95% bias-corrected confidence intervals for direct and indirect effects were generated based on 10,000 bootstrapped samples. However, as expected from the correlation analyses, no mediation was observed.

## Discussion

Subjective socioeconomic status, education and memory were the most important predictors of GHPS in the multivariate model. Although SSES was highly correlated with income and moderately correlated with education, the predictive effect of SSES was independent of objective economic (income) and intellectual (education and cognitive abilities) resources. Although SSES predicted GHPS, it should be taken into account that SSES was measured by making reference to the participant’s education, income and job. Hence we do not specifically how SSES reflects the plethora of psychological consequences related to being low in the socioeconomic hierarchy. This testifies to the importance of including SSES in future research on social disparities in health behaviours. Although the psychological mechanism linking SSES with health behaviours remains to be delineated, it has been suggested that being comparatively low in social status is related to reduced perceptions of power, resources, protection, popularity, and future opportunities (Sapolsky, 2005; Kraus et al., 2012; Haushofer and Fehr, 2014; Ishii et al., 2017). In such a situation, one may be better-off prioritising immediately available and certain opportunities and rewards (Bickel et al., 2014; Sheehy-Skeffington, 2018). Here, we hypothesised that the effect of SSES would be mediated by personal control perceptions, since low control would likely support more short-sighted decisions and behaviours, but this was not the case. At present, we are unable to shed light on other potential causal mechanisms. We suggest three promising avenues for future research. First, people with different socioeconomic positions, to a large extent, live in different neighbourhoods and are differently exposed to environmental-level facets, such as air and noise pollution, poor housing, traffic, criminality, drug use, walkability, availability of sports facilities, and availability of unhealthy foods, which likely influences health-related decisions and behaviours at the individual level (Schüz, 2017; McGowan and Shahab, 2019). Therefore, it has been suggested that researchers more often include data on ecological or area level (postal code, school district, census data, etc.) (Galobardes et al., 2006b; Schüz, 2017). It is possible that SSES better captures the psychological consequences of living under such circumstances, compared with the traditional indicators of OSES. Second, it is possible that SSES may be better than OSES indicators in reflecting membership in social classes. Social and cultural characteristics of the social classes seem to drive differences in social identity processes and self-construal, which influence various choices and behaviours (Manstead, 2018; Easterbrook et al., 2019). Third, it is possible that SSES captures the psychological consequences of social defeat better than OSES indicators. Social defeat implies that the individual perceives a failed struggle related to loss of status, identity, or resources (Selten et al., 2013). Modern societies are characterised by high levels of social competition, and social defeat in humans can arise from a lack of perceived ability to compete in everyday life (Carvalho et al., 2013). In fact, in a rich society with substantial inequality, the feeling of being poor and low in the socioeconomic hierarchy may be particularly salient for self-perceptions and personal identity (Cheung and Lucas, 2016; Payne, 2017). This may contribute to explaining why there seems to be a gradient effect of SES, for many outcomes, even in rich countries (Noble et al., 2007; Huijts et al., 2010; McGowan and Shahab, 2019). It is possible that SSES, beyond OSES, captures the psychological consequences of social defeat. In summary, the present study supported the possibility that SSES captures the psychological consequences of living under more harsh and unfriendly circumstances in a socioeconomic hierarchy, more broadly than OSES indicators. In short, SSES and OSES may relate differently to the social context, which has indeed been suggested as a potential explanation for why they are often found to correlate only moderately (Destin et al., 2017).

The current study showed that SSES and income, but not education, were related to PM and PC. Such findings are in accordance with studies that have demonstrated a link between the perception of being low on the social hierarchy and perceptions of personal control (Sheehy-Skeffington, 2019). Supplementary analyses (multiple regression, not reported) showed that income was a stronger predictor than SSES of both PC and PM. Hence, economic resources seem to be important for the control that people perceive to have over their daily life trade-offs. However, while previous research has demonstrated that people who believe they have control over important elements of their lives are generally more likely to engage in health-promoting behaviours and less likely to engage in health-threatening behaviours (Pepper and Nettle, 2014a; Sheehy-Skeffington, 2018), this was not observed in the present study. Hence, control perceptions were associated with SSES and income, but not with GHPS, and personal control measures did not mediate between SSES or education and GHPS.

In the current study, income and education were not significantly associated. This finding is in accord with the notion that although OSES may be a convenient summary term, it should probably not be conceived of as a unidimensional construct. Rather, each OSES indicator may emphasise a particular aspect of social stratification (Galobardes et al., 2007). This reasoning has been explicitly acknowledged in epidemiological health research, probably due to the fact that different OSES indicators exert unique causal effects on health (e.g., different types of occupational hazards) (Geyer et al., 2006; Galobardes et al., 2007). However, such awareness seems to be less prominent in health behaviour research, where OSES indicators often seem to have been summed or used interchangeably, or to have been selected with little explicit theoretical reasoning. This may have hindered revealing the specific psychological mechanisms which may potentially mediate between socioeconomic position and decisions and behaviours. The results of the current study showed that education and income were related differently to GHPS, in that education, but not income, predicted GHPS. This finding is in accordance with the results of previous research. Hence, generally, education seems to be a more important predictor of health behaviours than income, and the effect of income on health behaviours tends to be substantially attenuated when adjusting for the effect of education (Laaksonen et al., 2008). Such observations relate to the reasoning of Pepper and Nettle (2014b) that some health behaviours are not related to financial costs at all (e.g., sleep, self-examination, and health communication), while in other cases, the more healthy behavioural option is the less costly one (e.g., limiting coffee, tobacco, and alcohol use). Importantly, such reasoning and our findings do not necessarily imply that lack of money does not directly impact specific health behaviours, although a linear association between income and health behaviours was not observed. Indeed, many decisions and behaviours may stem simply from having little money (Shah et al., 2012). For example, eating healthy foods may be unaffordable for many families and individuals, and in England, the poorest 10 percent of households would need to spend close to three-quarters of their disposable income on food to meet the national dietary guidelines, compared with only six percent of the household income in the richest decile (Food Foundation, 2021). In sum, it seems fair to assume that the direct causal effect of purchase ability may be more important in those who are poor and that it may vary over different health behaviours. Laaksonen et al. (2008) suggested that income may work as a general socioeconomic indicator that is related to other socioeconomic indicators. A bivariate relationship between income and health behaviours may reflect that income accounts for a substantial proportion of the effects of all other socioeconomic variables related to health behaviours, but which are not measured or adjusted for in the analysis. However, this explanation does not seem to account for our findings. Income was substantially related to SSES but not to education. More importantly, no bivariate association was observed between income and the GHPS. At first glance, these findings may appear to be at odds with the resource scarcity explanation, which emphasises the importance of absolute poverty or economic resource weakness to explain socioeconomic differences in decisions and behaviours in various areas of daily life (see Reimers et al., 2009; Haushofer and Fehr, 2014). However, before jumping to such a conclusion, it is important to keep in mind that lower income levels were associated with deflated PM perceptions and inflated PC perceptions in the present study (and these associations were upheld in supplementary analyses in terms of multivariate regression models controlling for SSES and education). Accordingly, it may be that income, for example, via personal control perceptions, influences various daily life trade-offs that depend on economic resources. However, the present study indicated that this was not the case for summary measures of health behaviours since neither income nor control perceptions were associated with GHPS. Notably, a different pattern was observed for education. While education had a bivariate association with GHPS and predicted GHPS in the multivariate model, education was not associated with personal control perceptions. In sum, the present study indicated that education and economic resources influence people’s daily life trade-offs differently and via different mechanisms. Consequently, it is important in future research to disentangle how and why different OSES socioeconomic indicators are causally related to various health behaviours (Galobardes et al., 2007; Pampel et al., 2010).

Not only were the effects of education and SSES on health behaviours independent of economic resources, but they were also independent of cognitive resources. Hence, none of the measures of cognitive abilities confounded the relationships between SSES and education and GHPS. Unexpectedly, intelligence and memory correlated negatively with GHPS, while attention did not correlate with GHPS. Moreover, the negative effect of memory on GHPS was upheld in the multivariate model, while the direct effect of intelligence did not reach significance in that model. Additionally, we did not observe any associations between any of the measures of cognitive abilities (intelligence, memory, and attention) and education or SSES. These findings were unexpected and at odds with much of the previous research. Several studies have reported that higher levels of intelligence predict healthy behaviours positively (Wraw et al., 2018; Deary et al., 2019) and that associations between SES indicators and health behaviours tend to be reduced or become non-significant when controlling for the effect of intelligence (Ciarrochi et al., 2012; Deary et al., 2019). Moreover, Gray-Burrows et al. (2019) and Reimann et al. (2020) concluded from a literature review and meta-analysis that higher EF tends to be associated with more health-promoting behaviours. Finally, the finding that none of the indicators of cognitive abilities in the current study correlated with education was at odds with previous research that has reported intelligence to consistently predict educational attainment (Marks, 2013; Plomin and von Stumm, 2018; Deary et al., 2021). We are not able to explain such discrepancies between the present study and previous research. We carefully checked the coding of the variables, and independent researchers who ran the analyses obtained the same results. We cannot rule out the possibility that the results of the current study are reflective of the characteristics of the sample or caused by methodological flaws. However, in such a context, it deserves to be mentioned that the methodological aspects of crowd-working platforms, including Prolific, for research purposes have been extensively investigated. The general finding is that such platforms can be a reliable source of high-quality and representative data for multiple research purposes in behavioural sciences (Crump et al., 2013; Simcox and Fiez, 2014; Hauser and Schwarz, 2016; Palan and Schitter, 2018). Moreover, in many ways, the present study seems to compare relatively well, methodologically, with previous relevant research on EF and health behaviours. For example, data on cognitive abilities are not based on self-reports. We included numerous tests of cognitive abilities, which we have considerable experience in using, and the tests correlated with each other as expected. As far as sample characteristics are concerned, we had a comparatively large sample and did not recruit participants from a specific risk group (e.g., patients, students, low-income, and homeless), which was the case in many previous studies (Reimann et al., 2020). Finally, the present sample comprised participants from all socioeconomic classes, and generally participants recruited from crowd-working platforms seem to be more diverse than, for example, college samples or online convenience samples (Stewart et al., 2017). Hence, the range of SES was not restricted, and the power to detect the expected associations between SES and cognitive abilities did not seem to be hindered. On the other hand, compared with representative samples, participants from crowd-working platforms tend to be more educated and report lower income and higher unemployment (Stewart et al., 2017). More importantly, crowd-working participants seem to have a different psychological profile than other samples. For example, they tend to score higher on learning goal orientation, need for cognition, introversion, neuroticism, and autism spectrum disorder (Stewart et al., 2017). However, we do not know exactly how such factors would contribute to explaining the unexpected results of the present study concerning the associations between cognitive abilities on the one side and GHPS and education on the other.

The present study had several limitations. First, the study was cross-sectional, which precludes any causal conclusions. Second, due to economic constraints, the sample was restricted to 200 participants. Third, the study took almost an hour to complete. It is possible that some individuals with attentional issues may have dropped out from completing the study, thus biasing the sample toward highly motivated individuals (or those who urgently needed the money paid as compensation for completing the survey). Fourth, health behaviours were measured using only self-report measures. Finally, SES measures were restricted to individual level measures; no structural or area level measures were included. To the extent that the results of the present study are genuine, they tend to accord with Clouston et al. (2015) in that socioeconomic factors (education and SSES in our study), but not cognitive abilities, explain why individuals who are low in the social hierarchy practice more unhealthy behaviours [for a discussion on this point, see also Deary and Johnson (2010) and Deary et al. (2021)]. However, such reasoning is not consistent with many of the studies included in the reviews and meta-analysis described above (Gray-Burrows et al., 2019; Reimann et al., 2020), in which cognitive abilities predicted health behaviours even after controlling for education.

## Conclusion and Implications

This study showed that a person’s position in the socioeconomic hierarchy matters for health behaviours. Being comparatively low in the socioeconomic hierarchy was related to practicing less health-promoting and more health-threatening behaviours. As hypothesised, SSES predicted health behaviours beyond the OSES indicators. Among the OSES indicators, education, but not income, predicted health behaviours. Contrary to our hypotheses, the effects of SSES and education on health behaviours were not attenuated when controlling for cognitive abilities, and their effects were not mediated by personal control. Unexpectedly, cognitive abilities were negatively associated with health-promoting behaviours.

Individuals’ SSES is likely influenced by perceived subjective inequality, that is, how much inequality individuals perceive there is within their society (Schmalor and Heine, 2021). It seems fair to suggest that subjective inequality and SSES contribute to explaining why SES has a gradient effect on many outcomes, including health behaviours, in rich countries. Importantly, although low income and lack of purchase ability may not necessarily exert a direct causal effect on (all) health-related decisions and behaviours in individuals living in affluent countries, objective income inequalities and subjective inequality may contribute to disparities in SSES, which may have important psychological consequences. If this reasoning is correct, it paves the way for policy development and interventions that are effective in reducing objective economic inequality, since such policy measures could potentially reduce subjective inequality. First, a redistribution of income and wealth may prove effective and should be a primary target in effective policies (Piketty, 2013). Second, the most effective interventions are likely those that provide high-quality education to all children (Conti et al., 2010; Deary et al., 2021). This is supported by health behaviour research, including the current study, demonstrating a link between low education and increased practice of unhealthy behaviours. Third, it should be taken into consideration that socioeconomic characteristics at the ecological level may interact with individual factors that influence health behaviours (Schüz, 2017). However, while many intervention studies have focused on indicators at the individual level (e.g., knowledge, attitudes, and control beliefs), few studies have examined the effects of interventions at the ecological or structural level (changing neighbourhood characteristics such as pollution, criminality, and walkability) (Schüz, 2017). One interesting study indicating the efficacy of such policy measures was conducted by Liu et al. (2012), who reported that including subtle contextual features of poverty in local living conditions could influence intertemporal choices. This suggests that interventions that change situational cues of poverty in local environments can shift intertemporal choices and support more healthy choices independent of individual characteristics.

## Data Availability Statement

The raw data supporting the conclusions of this article will be made available by the authors, without undue reservation.

## Ethics Statement

The studies involving human participants were reviewed and approved by Department of Psychology, University of Oslo Research Ethics Committee (Ref. number: 6366060). The patients/participants provided their written informed consent to participate in this study.

## Author Contributions

PK and TE conceived of the presented idea. PK, TE, and TH developed the questionnaires. TH collected the data and performed the computations. PK and BK performed the initial data analysis. TE and TH verified the analysis. PK developed the first draft. All authors commented and contributed to the final manuscript.

## Conflict of Interest

The authors declare that the research was conducted in the absence of any commercial or financial relationships that could be construed as a potential conflict of interest.

## Publisher’s Note

All claims expressed in this article are solely those of the authors and do not necessarily represent those of their affiliated organizations, or those of the publisher, the editors and the reviewers. Any product that may be evaluated in this article, or claim that may be made by its manufacturer, is not guaranteed or endorsed by the publisher.

## Footnotes

1
prolific.co
